# Supplementary material for: Signatures of a magnetic-field-induced Lifshitz transition in the ultra-quantum limit of the topological semimetal ZrTe5
Source: Nat Commun. 2022 Dec 1;13:7418. doi: 10.1038/s41467-022-35106-7 (PMC9715529; doi:10.1038/s41467-022-35106-7)
Supplement: Supplementary file 1 — Supplementary Information [file 41467_2022_35106_MOESM1_ESM.pdf]

Supplementary Information

Signatures of a magnetic-field-induced Lifshitz transition in the ultra-quantum limit of the topological semimetal $ZrTe_5$

S. Galeski[1, 2*], H.F. Legg[3], R. Wawrzyńczak[1], T. Förster[4], S. Zherlitsyn[4], D. Gorbunov[4], M. Uhlarz[4], P. M. Lozano[5], Q. Li[5], G.D. Gu[5], C. Felser[1], J. Wosnitza[4,6], T. Meng[7], J Gooth[1,2#]

[1]Max Planck Institute for Chemical Physics of Solids, Nöthnitzer Straße 40, 01187 Dresden, Germany.

[2]Physikalisches Institut, Universität Bonn, Nussallee 12, D-53115 Bonn, Germany

[3]Department of Physics, University of Basel, Klingelbergstrasse 82, CH-4056, Basel, Switzerland

[4]Hochfeld-Magnetlabor Dresden (HLD-EMFL) and Würzburg-Dresden Cluster of Excellence ct.qmat, Helmholtz-Zentrum Dresden-Rossendorf, 01328 Dresden, Germany

[5]Condensed Matter Physics and Materials Science Department, Brookhaven National Laboratory, Upton, NY, USA.

[6]Institut für Festkörper- und Materialphysik, Technische Universität Dresden, 01069 Dresden, Germany

[7]Institute of Theoretical Physics and Würzburg-Dresden Cluster of Excellence ct.qmat, Technische Universität Dresden, 01069 Dresden, Germany

***stanislaw.galeski@cpfs.mpg.de, [#] johannes.gooth@cpfs.mpg.de**

S1 Charge-carrier density and mobility from Hall-effect measurements

From linear fits to the low-field Hall data (Supplementary Fig. S2a and b), we obtain the temperature-dependent dominant charge-carrier concentration $n_{\mathrm{hall}} = (\mathrm{d}\rho_{\mathrm{xy}}/\mathrm{d}|B|\cdot e)^{-1}$ and the average mobility $\mu_{\mathrm{hall}} = (\rho_{\mathrm{xx,0}}\, en)^{-1}$ of $ZrTe_5$, using a single-band model[1]. The smaller charge-carrier density of Sample C with the quantum limit occurring at 0.6 T is additionally confirmed by the lower field of the Lifshitz transition as seen in Supplementary Fig. S1 [2]

S2 Determination of cyclotron mases

The amplitude of the Shubnikov-de Haas oscillations in the oscillatory part of the longitudinal electrical resistivity $\Delta\rho_{\mathrm{xx}}(\boldsymbol{B})$ is proportional to $\chi(\boldsymbol{B})/\sinh[\chi(\boldsymbol{B})]$ with the cyclotron frequency $\omega_{\mathrm{c},j} = \frac{e\,|\boldsymbol{B}|}{m_{c,j}}$ and $\chi(\boldsymbol{B}) = \frac{2\pi^2 k_{\mathrm{B}} T m_{\mathrm{c}}}{\hbar e|\boldsymbol{B}|}$ here $m_{\mathrm{c},j}$ is the cyclotron mass. $\Delta\rho_{\mathrm{xx}}(\boldsymbol{B})$ is obtained from subtracting a smooth background from the measurement data using a second-order polynomial. The corresponding effective mass can be extracted from fitting $\chi(\boldsymbol{B})/\sinh[\chi(\boldsymbol{B})]$ to the T-dependent amplitude[3] (Supplementary Fig. S7f-h).

S3 Calculation of the Hall conductivity tensor

We calculate the Hall conductivity tensor element σ_{xy} using $\sigma_{xy} = \rho_{xy}/(\rho_{xx}^2+\rho_{xy}^2)$, assuming that $\rho_{xx} = \rho_{yy}$. However, in general $\sigma_{xy} = \rho_{xy}/(\rho_{xx}\,\rho_{yy} + \rho_{xy}^2)$ with a magnetic field in b-direction. Due to the geometry of the $ZrTe_5$ crystals (elongated needles) and its mechanical fragility, performing reliable measurements of ρ_{yy} on our samples is not possible as described in ref 4.

However, in our previous study of the magnetoresistance of $ZrTe_5$ single crystals we have estimated that $\rho_{yy}/\rho_{xx} \approx 0.8$ and thus the error in assuming $\sigma_{xy} = \rho_{xy}/(\rho_{xx}^2+\rho_{xy}^2)$ is small[4,5].

S4 Comparison of charge carrier densities obtained from Hall measurements and the volume of the Fermi Surface

Since fits to the Hall effect are performed in a narrow field window to avoid the influence of quantum oscillations that appear already at 0.1T, they could be prone to a systematic error. As a secondary test we have extracted the charge carrier density by integration of the fermi surface volume assuming and elliptical Fermi surface[5]. We have extracted the FS cross-sections $S_{F,j}$ using the standard Onsager relation $B_{F,j} = (\hbar/2\pi e)S_{F,j}$ where $B_{F,j}$ is the SdH frequency. SdH frequencies along the principal crystal axis were extracted through analysis of Landau fan diagrams, see Figure S10 and S11. Using the standard relations between ellipse cross-sections and radii we have extracted the relevant Fermi wave vectors and estimated the charge carrier density from the FS volume according to the equation:

$$\boldsymbol{n_{SdH} = \frac{1}{(2\pi)^3}\frac{4}{3}\pi k_{f,a}k_{f,b}k_{f,c}} \quad (1)$$

The charge densities obtained from Hall fits and FS volume estimates are in reasonable agreement, see table S1.

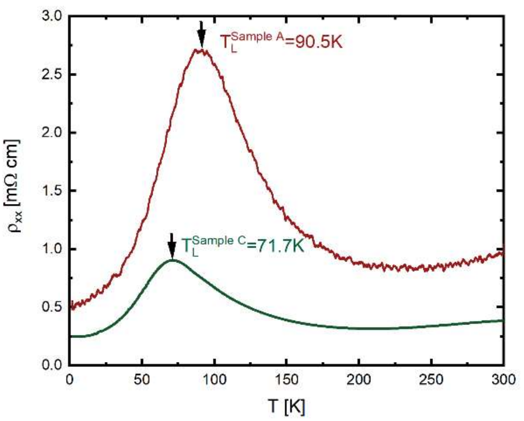

Supplementary Fig. S1. Longitudinal resistivity ρ_{xx} of $ZrTe_5$ Sample A and C as a function of temperature *T* at zero magnetic field.

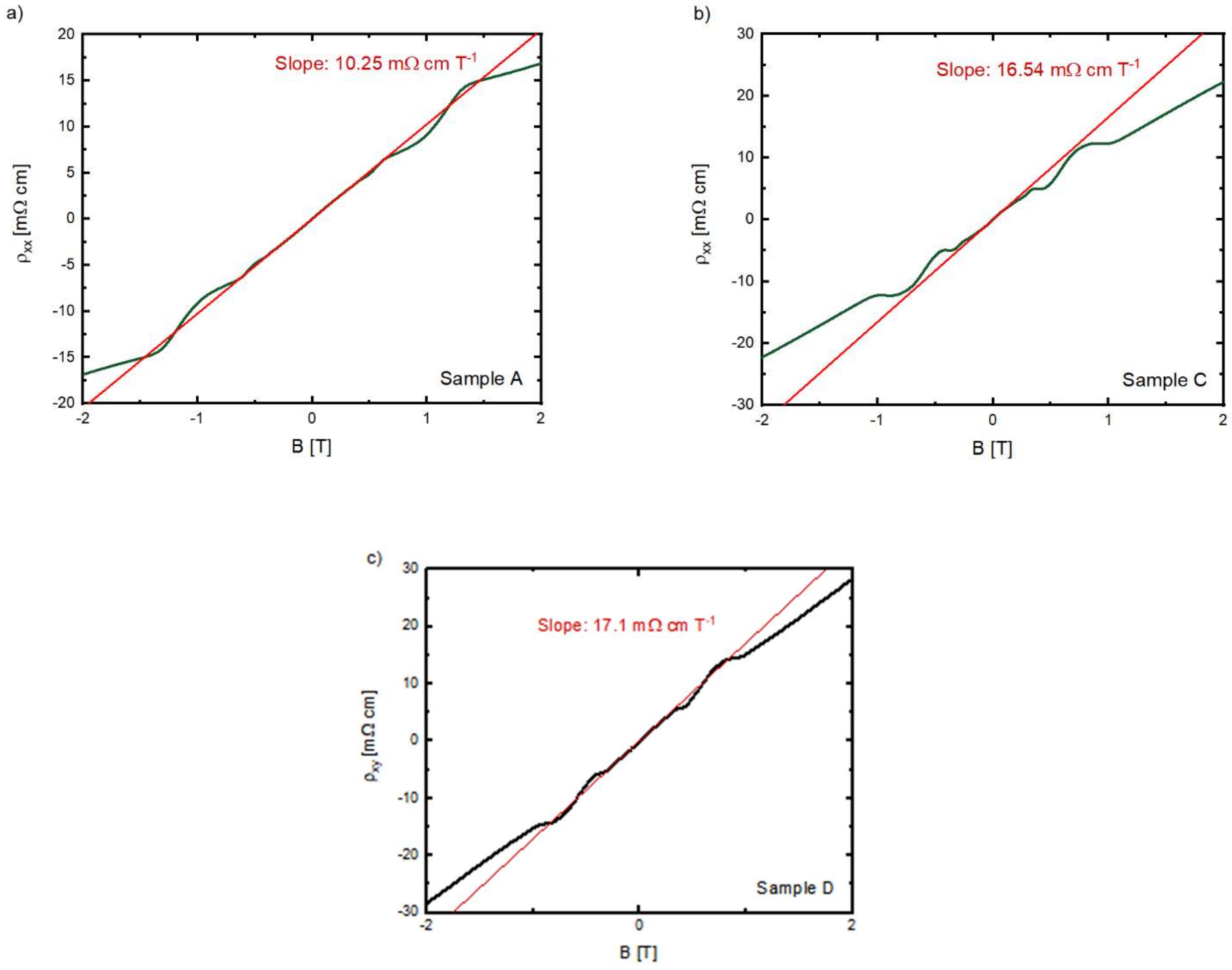

Supplementary Fig. S2. Linear fits of the Hall resistivity at low magnetic fields: a. Sample A and **b.** Sample C, **c.** Sample D. Black curves represent measured Hall resistivities. Red curves represent linear fits to ρ_{xy} using data in the range ±0.1 T.

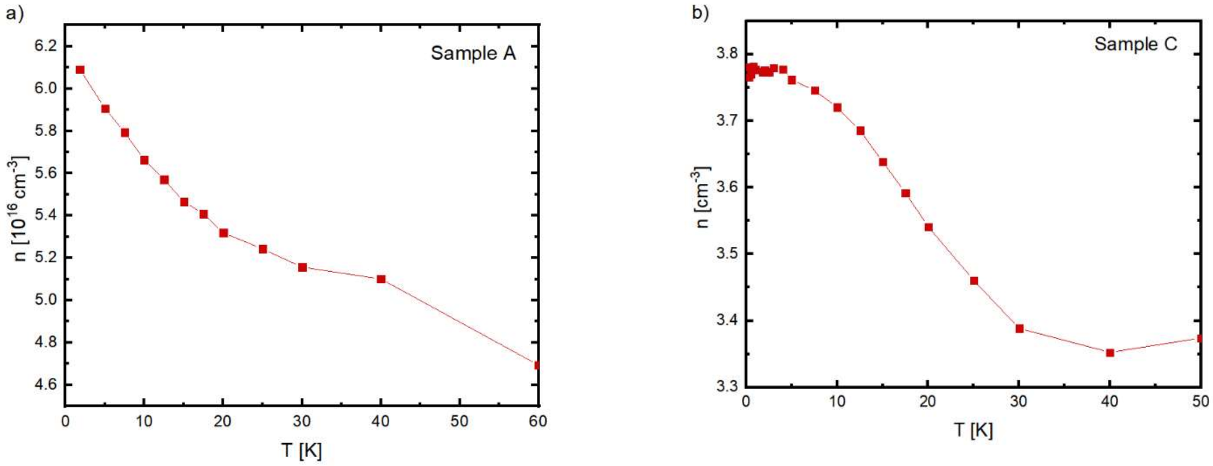

Supplementary Fig. S3. Charge-carrier concentration as a function of temperature. a. Sample A and **b.** Sample C. Charge-carrier concentration $n = (d\rho_{xy}/d|\boldsymbol{B}|\cdot e)^{-1}$ is extracted from the slope of the linear fits to the Hall resistivity.

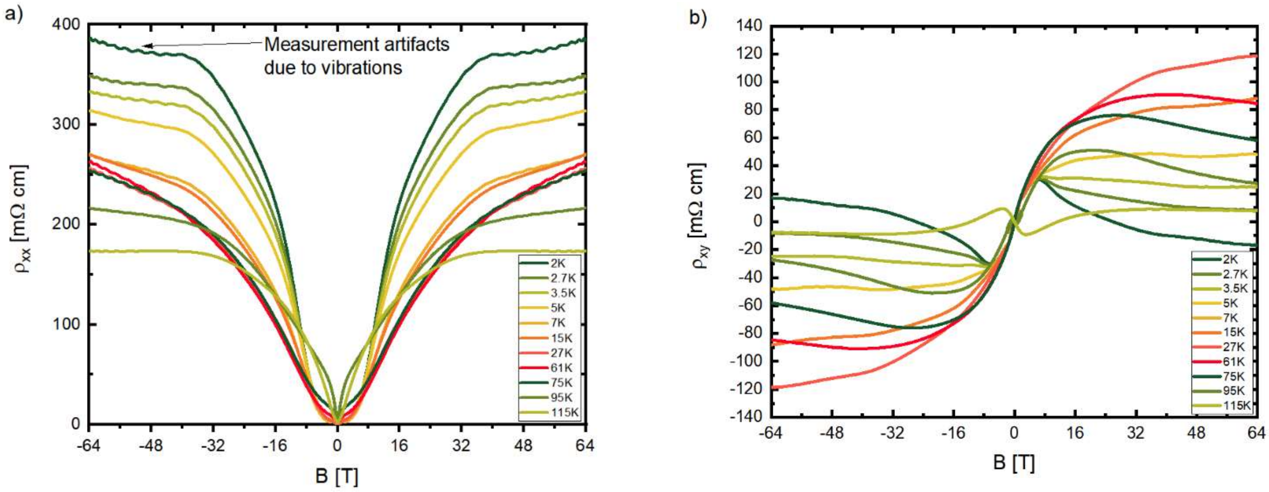

Supplementary Fig. S4. Longitudinal electrical resistivity ρ_{xx} and the Hall resistivity ρ_{xy} of Sample A at various temperatures. a, ρ_{xx} and **b**, ρ_{xy} as a function of magnetic field *B* for various *T* with *B* applied along the *b*-axis. The measurements were performed in pulsed magnetic fields. Additional oscillations seen at high fields above 32 T represent high-frequency vibrations of the sample assembly during the pulse.

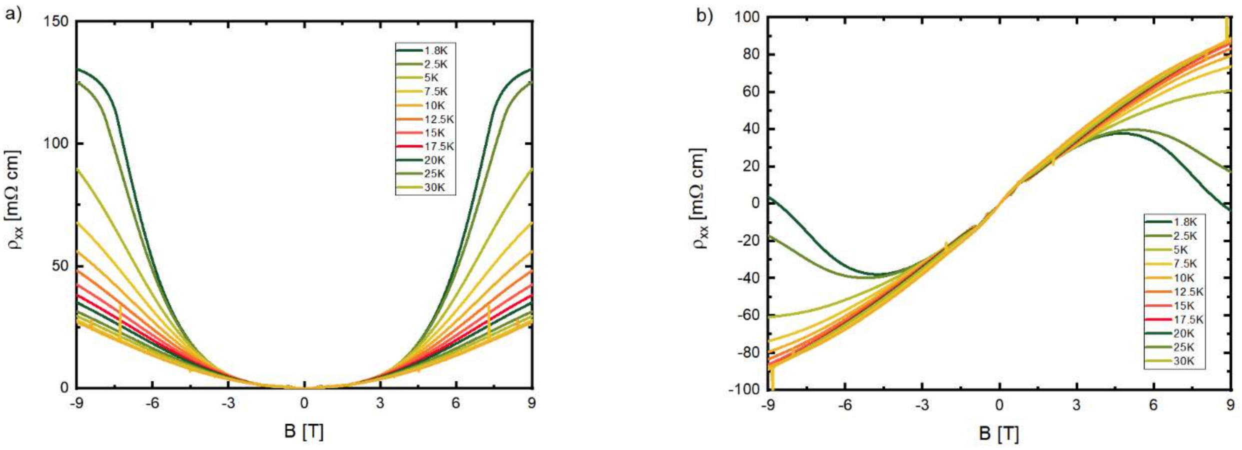

Supplementary Fig. S5. Longitudinal electrical resistivity ρ_{xx} and the Hall resistivity ρ_{xy} of Sample C at various temperatures. a, ρ_{xx} and **b**, ρ_{xy} as a function of magnetic field B for various T with B applied along the b-axis. The measurements were performed in a cryostat equipped with a 9 T DC magnet.

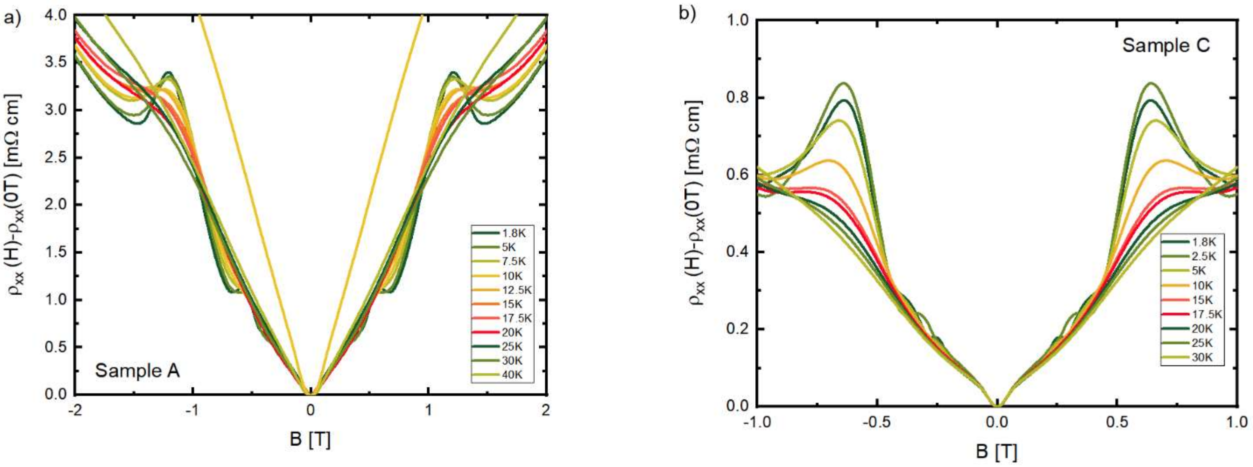

Supplementary Fig. S6. Low-field longitudinal electrical resistivity ρ_{xx} at various temperatures. a. Sample A and **b.** Sample C measured as function of magnetic field B for various T with B applied along the b-axis. The measurements were performed in a cryostat equipped with a 9 T DC magnet. (The labels (R_{xx}) are wrong).

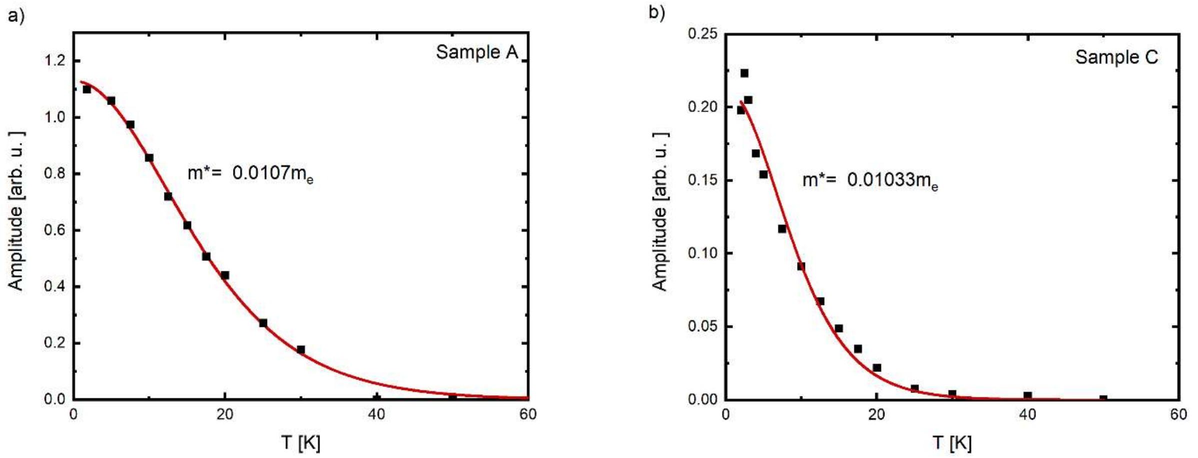

Supplementary Fig. S7. Determination of the cyclotron mass for *B* applied along the crystallographic *b*-axis. **a.** Fit of the Lifshitz-Kosevitsh formula to the temperature dependence of the amplitude of the n = 1 quantum oscillation of sample A **b.** Fit of the Lifshitz-Kosevitsh formula to the temperature dependence of the amplitude of the n = 1 quantum oscillation of sample C.

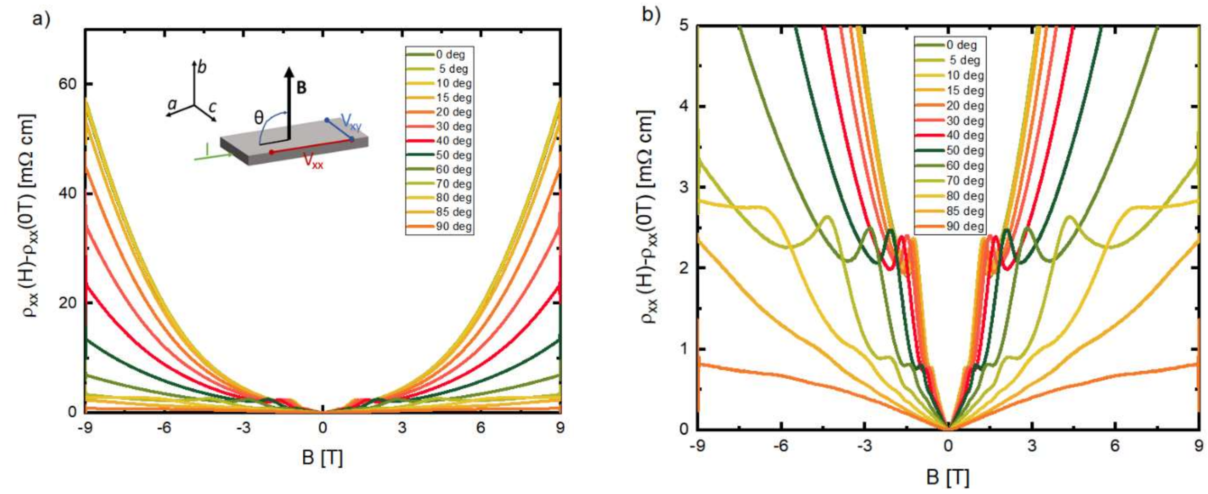

Supplementary Fig. S8. Angular dependance of magnetoresistance of sample A. Magnetoresistance versus magnetic field B for various rotation angles in the a-b plane, measured at 1.8K.

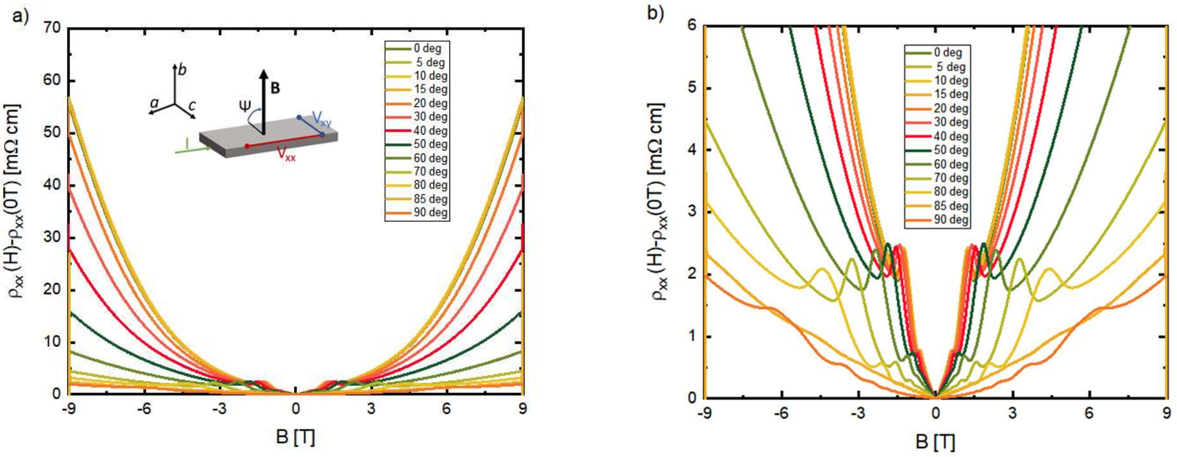

Supplementary Fig. S9. Angular dependance of magnetoresistance of sample A. Magnetoresistance versus magnetic field B for various rotation angles in the b-c plane, measured at 1.8K.

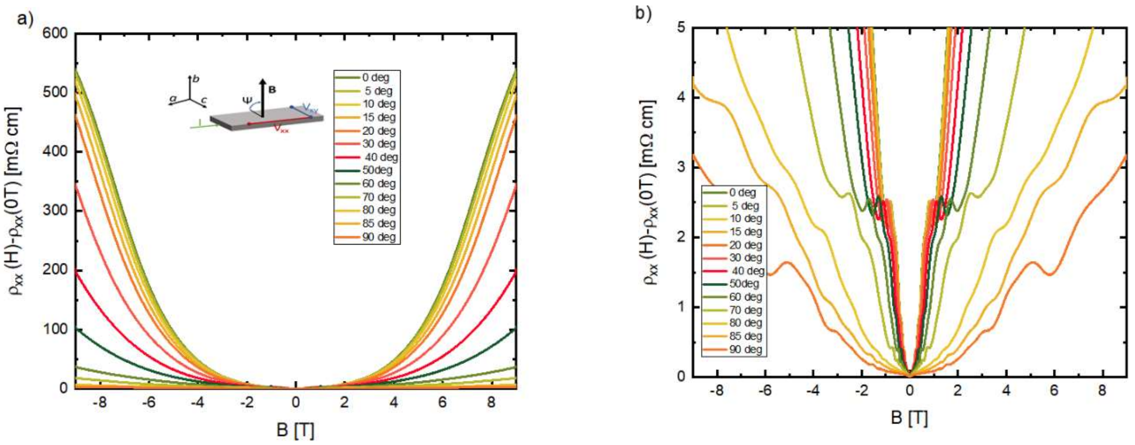

Supplementary Fig. S10. Angular dependance of magnetoresistance of sample D. Magnetoresistance versus magnetic field B for various rotation angles in the b-c plane, measured at 1.8K.

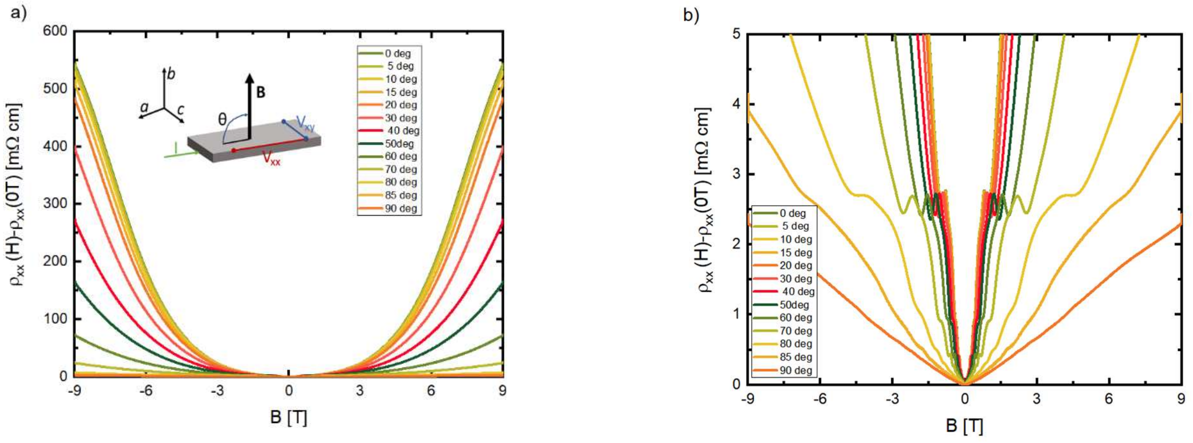

Supplementary Fig. S11. Angular dependance of magnetoresistance of sample D. Magnetoresistance versus magnetic field B for various rotation angles

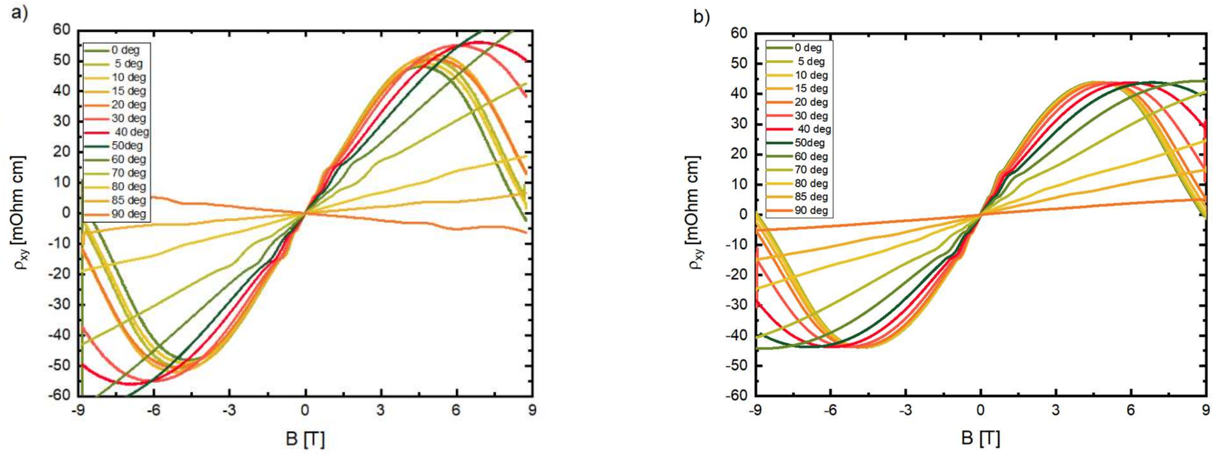

Supplementary Fig. S12. Angular dependance of Hall effect in sample D. Magnetoresistance versus magnetic field B for various rotation angles in the **a.** a-b plane **b.** c-b plane, measured at 1.8K.

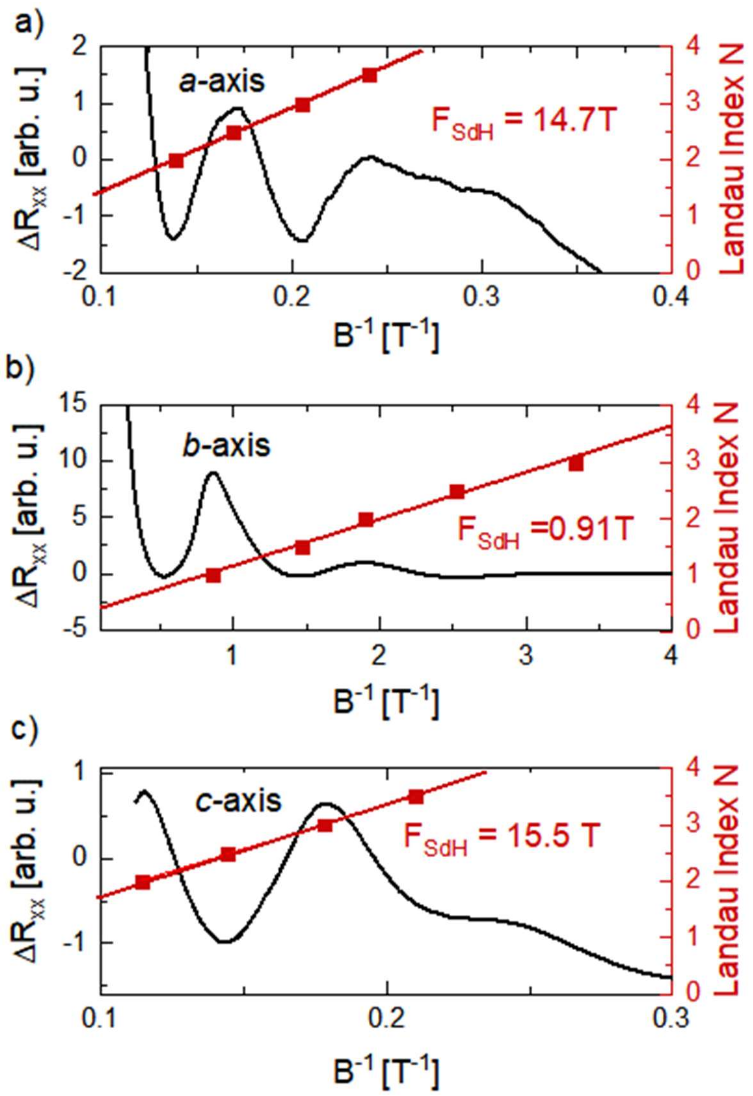

Supplementary Fig. S11. Analysis of quantum oscillations of sample A. a) Oscillatory part of the magnetoresistance obtained with B||a and J||a and the corresponding Landau fan diagram. **b)** Oscillatory part of the magnetoresistance obtained with B||b and J||a and the corresponding Landau fan diagram. **c)** Oscillatory part of the magnetoresistance obtained with B||c and J||a and the corresponding Landau fan diagram. ΔR was obtained by subtracting a second order polynomial from the data.

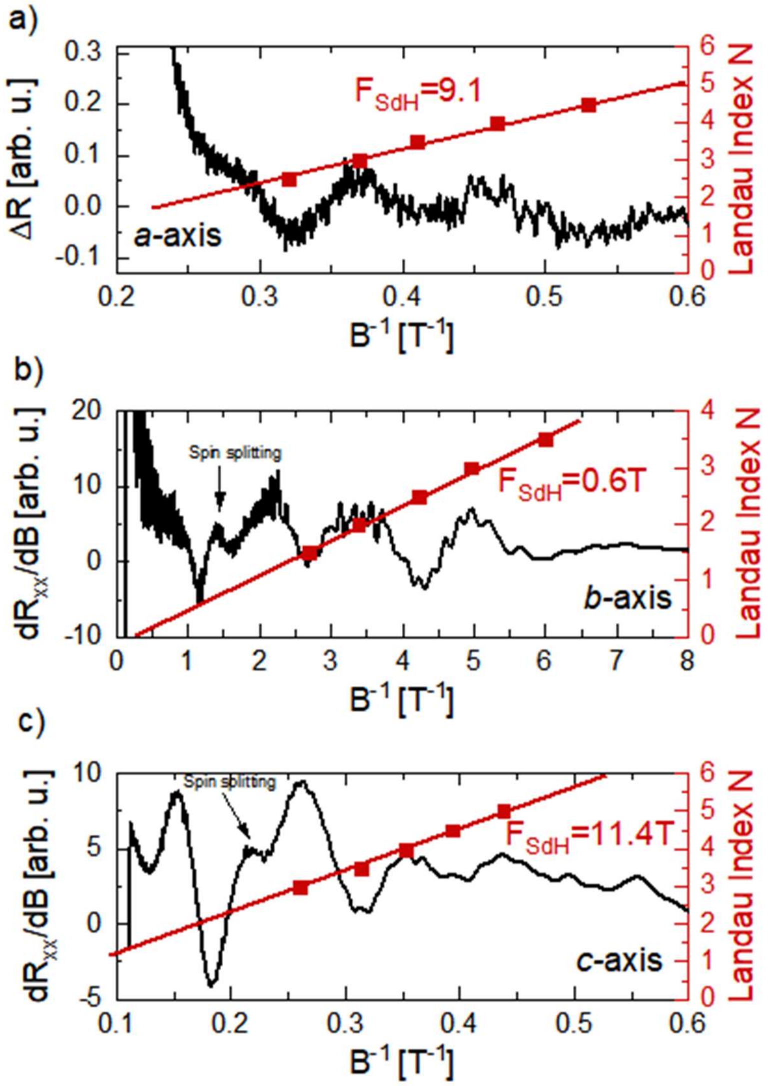

Supplementary Fig. S12. Analysis of quantum oscillations of sample A. a. Oscillatory part of the magnetoresistance obtained with B||a and J||a and the corresponding Landau fan diagram. **b.** Derivative of the oscillatory part of the magnetoresistance obtained with B||b and J||a and the corresponding Landau fan diagram. **c)** Derivative of the oscillatory part of the magnetoresistance obtained with B||c and J||a and the corresponding Landau fan diagram. ΔR was obtained by subtracting a second order polynomial from the data. Where subtraction of a reasonable background was difficult a derivative was taken. Points for the fan diagrams were determined only in the region where oscillations were clear and not yet spin-split. In the case of datasets where derivatives were taken fan diagrams reflect only feature location in field not real Landau level indices.

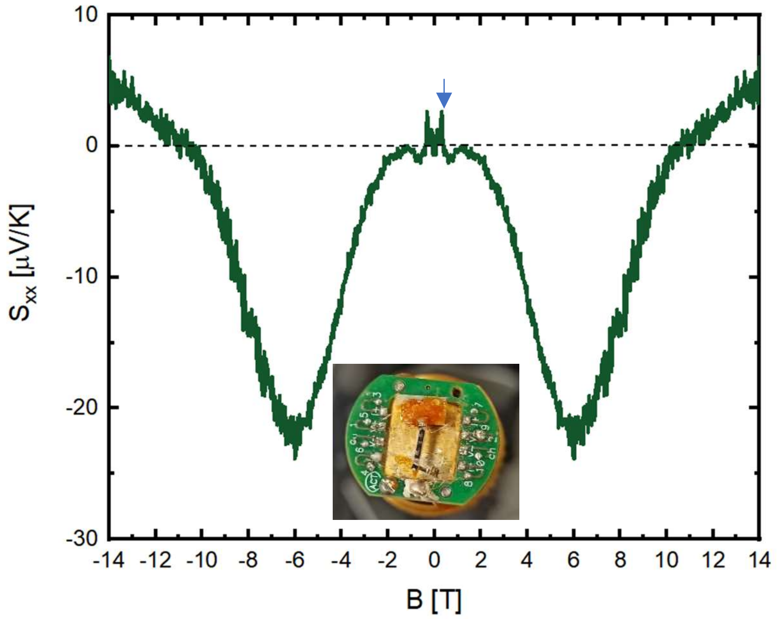

Supplementary Fig. S12. Field dependance of the Seebeck effect measured in sample D at 550mK. The data was taken with magnetic field applied along the b-axis and thermal gradient along the a-axis. The thermal gradient was set not to exceed 10% of the average sample temperature ~ ca 50mK. The temperature gradient across the sample was measure using Cernox CX-1010 thermometers glued directly to the Voltage contacts. In order to remove the effect of thermal voltage buildup across the measurement wires a background signal was measured and subtracted from the measured value. The inset presents the sample on the PPMS He3 sample puck. The sample was installed semi-suspended in order to allow a measurable gradient buildup. The presented data was symmetrized to exclude remove the asymmetric component originating from the Nernst effect. The arrow marks measurement artifacts that we attribute to a brief cryostat temperature instability due to the magnetic field reversal.

	Axis	B_F [T]	$S_{F,j}$ [m^{-2}]	k_F [cm^{-1}]	n_{hall} [cm^{-3}]	n_{SdH} [cm^{-3}]
Sample A	a	14.7	$14\cdot10^{17}$	$5.4\cdot10^{5}$	$6.1\cdot10^{16}$	$4.1\cdot10^{16}$
	b	0.91	$8.7\cdot10^{15}$	$87\cdot10^{5}$		
	b	15.5	$1.5\cdot10^{17}$	$5.1\cdot10^{5}$		
Sample D	a	9.1	$8.7\cdot10^{16}$	$4.8\cdot10^{5}$	$3.9\cdot10^{16}$	$2.2\cdot10^{16}$
	b	0.6	$5.7\cdot10^{15}$	$72\cdot10^{5}$		
	c	11.4	$1.1\cdot10^{17}$	$3.8\cdot10^{5}$		

Supplementary table S1: Fermi surface parameters of samples A and D

Supplementary References

1. Ashcroft, N. W. & Mermin, N. D. *Solid State Physics*. (Saunders College, 1976).

2. Tang, F. *et al.* Three-dimensional quantum Hall effect and metal–insulator transition in $ZrTe_5$. *Nature* **569**, 537–541 (2019).

3. Shoenberg, D. *Magnetic Oscillations in Metals*. (Cambridge University Press, 1984).

4. Galeski, S. *et al.* Unconventional Hall response in the quantum limit of $HfTe_5$. *Nat. Commun.* **11**, 5926 (2020).

5. Galeski, S. *et al.* Origin of the quasi-quantized Hall effect in ZrTe5. *Nat. Commun.* **12**, 3197 (2021).
